# SolEST database: a "one-stop shop" approach to the study of *Solanaceae *transcriptomes

**DOI:** 10.1186/1471-2229-9-142

**Published:** 2009-11-30

**Authors:** Nunzio D'Agostino, Alessandra Traini, Luigi Frusciante, Maria Luisa Chiusano

**Affiliations:** 1 University of Naples 'Federico II', Dept of Soil, Plant, Environmental and Animal Production Sciences, Via Università 100, 80055 Portici, Italy

## Abstract

**Background:**

Since no genome sequences of solanaceous plants have yet been completed, expressed sequence tag (EST) collections represent a reliable tool for broad sampling of *Solanaceae *transcriptomes, an attractive route for understanding *Solanaceae *genome functionality and a powerful reference for the structural annotation of emerging *Solanaceae *genome sequences.

**Description:**

We describe the SolEST database http://biosrv.cab.unina.it/solestdb which integrates different EST datasets from both cultivated and wild *Solanaceae *species and from two species of the genus *Coffea*. Background as well as processed data contained in the database, extensively linked to external related resources, represent an invaluable source of information for these plant families. Two novel features differentiate SolEST from other resources: i) the option of accessing and then visualizing *Solanaceae *EST/TC alignments along the emerging tomato and potato genome sequences; ii) the opportunity to compare different *Solanaceae *assemblies generated by diverse research groups in the attempt to address a common complaint in the SOL community.

**Conclusion:**

Different databases have been established worldwide for collecting *Solanaceae *ESTs and are related in concept, content and utility to the one presented herein. However, the SolEST database has several distinguishing features that make it appealing for the research community and facilitates a "one-stop shop" for the study of *Solanaceae *transcriptomes.

## Background

*Solanaceae *represents one of the largest and most diverse plant families including vegetables (e.g. tomato, potato, capsicum, and eggplant), commercial (e.g. tobacco) and ornamental crops (e.g. petunia). Some *Solanaceae *plants are important model systems such as tomato for fruit ripening [1,2], tobacco for plant defence [3], and petunia for the biology of anthocyanin pigments [4].

Since no full genome sequence of a member of the *Solanaceae *family is yet available, though genome sequencing efforts are at the moment ongoing for tomato [5], potato http://www.potatogenome.net/ and tobacco http://www.tobaccogenome.org/, much of the existing worldwide sequence data consists of Expressed Sequence Tags (ESTs). Because of the useful information these data bring to the genomics of *Solanaceae *plants, the availability of EST collections has dramatically increased in size, partly thanks to the start-up of sequencing initiatives [6]. EST collections are certainly no substitute for a whole genome scaffold. However, they represent the core foundation for understanding genome functionality, the most attractive route for broad sampling of *Solanaceae *transcriptomes and, finally, a valid contribution to comparative analysis at molecular level on the *Solanaceae *family members. ESTs are a versatile data source and have multiple applications which result from the specific analytical tools and methods accordingly used to process this type of sequence.

Therefore EST databases are useful not only to strictly serve as sequence repositories, but as powerful tools, albeit relatively under-exploited and far from complete.

Several web resources have been established for collecting ESTs and for improving and investigating their biological information content due to the growing interest in *Solanaceae *genomics research. Some of them, mainly focusing on individual species, address the needs of a particular research community by providing a catalogue of putative transcripts, describing their functional roles and enabling gene expression profiling [7,8]. The remaining represent data-gathering centres or rather comprehensive resources aiming to meet the challenges raised by the management of multiple information from diverse sources worldwide [9-11].

The ultimate goal of these gene index providers is to represent a non-redundant view of all EST-defined genes. The unigene builds, which emerge, serve as the basis for a number of analyses comprising the detection of full-length transcripts and potential alternative splicing, expression pattern definition, association to array probes and, as a consequence, to microarray gene expression databases; association to metabolic and signalling pathways; development of simple sequence repeat (SSR) and conserved ortholog set (COS) markers etc.

We present the SolEST database, which integrates different EST datasets from both cultivated and wild *Solanaceae *species and also two EST collections from *Rubiaceae *(genus *Coffea*). SolEST is built on the basis of a preceding effort which was centred on the investigation of ESTs from multiple tomato species [12]. The main purpose is corroborating the existing transcriptomics data which are part of the multilevel computational environment ISOL@ [13]. In addition, the *Solanaceae *EST-based survey can considerably contribute to genome sequence annotation by highlighting compositional and functional features. Indeed, SolEST is a valuable resource for the ongoing genome sequencing projects of tomato (*S. lycopersicum*) [5] and potato (*S. tuberosum*; http://www.potatogenome.net/) and has the potential to significantly improve our understanding of *Solanaceae *genomes and address sequence-based synteny issues.

A common complaint in the SOL community concerns the different unique transcript sets generated for a given *Solanaceae *species by diverse research groups. These worldwide resources [9-12] are built starting from different primary data sets and by applying diverse methods and user-defined criteria for sequence analysis. Of course, there are advantages and disadvantages associated to each set, but to our knowledge, there is currently no easy way to compare them and, as a consequence, to provide the scientific community with a comprehensive overview. To this end, we also propose, as a novel feature of the SolEST database, a combined resource/interface dedicated to enabling the combination of different unigene collections for each *Solanaceae *species based on the UniProt Knowledgebase annotations.

The collection and integration of the whole public dataset of *Solanaceae *ESTs facilitate a "one-stop shop" for the study of *Solanaceae *transcriptomes.

## Construction and content

### Sequence retrieval

EST sequences are downloaded from dbEST http://www.ncbi.nlm.nih.gov/dbEST/ and from the Nucleotide/mRNA division of GenBank (release 011008).

### EST/mRNA processing pipeline

The EST processing and annotation pipeline is described in [14] although it has been recently upgraded by updating the set of databases used in EST vector cleaning and repeat masking and in the annotation phase. In addition, the clustering tool was replaced with a more efficient novel method presented in [15]. This pipeline, divided into four consecutive steps, was used for processing EST data from 14 cultivated and wild *Solanaceae *species and from two species belonging to the genus *Coffea *(Table 1).

**Table 1:** The SolEST database statistics.

Source	# ESTs	EST length	# mRNA	# mRNA length	# Cluster	# TCs	TC length	# ESTs in TCs	# sESTs	SEST length	# Unique transcripts
**SOLLC**	259990	522.47 ± 156.39	5770	1377.23 ± 735.42	17001	20548	1019.43 ± 544.69	234297	30937	491.07 ± 246.90	51485
**SOLPN**	8346	460.38 ± 129.80	13	1854 ± 974.32	817	844	666.91 ± 265.85	5249	3110	470.86 ± 165.38	3954
**SOLHA**	8000	617.39 ± 165.42	30	864.07 ± 537.43	1119	1243	900.79 ± 342.79	5323	2707	561.14 ± 171.26	3950
**SOLLP**	1008	352.89 ± 133.45	-	-	103	109	478.06 ± 151.54	413	594	342.03 ± 136.70	703
**RNA**	231275	611.41 ± 205.52	1704	1144.24 ± 663.38	18590	23453	983.56 ± 429.02	184233	48630	627.26 ± 236.38	72083
**SOLCH**	7752	812.65 ± 152.46	60	1008.97 ± 533.89	632	637	845.18 ± 266.09	1513	6279	824.93 ± 154.44	6916
**TOBAC**	240440	601.54 ± 231.81	3605	795.75 ± 805.60	24274	28571	934.99 ± 423.1	158264	81247	565.44 ± 262.19	109818
**NICBE**	42566	611.86 ± 243.81	301	1260.82 ± 1379.73	4452	5006	984.41 ± 443.85	29051	13784	505.82 ± 299.38	18790
**NICSY**	8583	381.48 ± 168.21	94	1577.17 ± 1125.60	662	674	457.99 ± 437.09	1838	6831	400.25 ± 209.85	7505
**NICAT**	329	303.60 ± 152.31	94	1239.71 ± 726.51	32	32	949.5 ± 775.84	68	352	461.88 ± 463.58	384
**NICLS**	12448	492.11 ± 205.98	95	831.58 ± 456.42	1268	1379	651.89 ± 252.34	7570	4969	467.14 ± 215.44	6348
**CAPAN**	33311	466.73 ± 154.98	564	974.95 ± 587.75	4082	4293	760.45 ± 331.46	22144	11714	460.65 ± 194.94	16007
**CAPCH**	372	464.35 ± 228.64	105	1072.8 ± 642.35	32	34	901.97 ± 490.54	86	389	572.65 ± 446.71	423
**PETHY**	14017	500.50 ± 185.75	323	1254.12 ± 724.09	1627	1738	704.4 ± 308.88	6642	7612	520.40 ± 268.87	9350
**COFCA**	55694	613.87 ± 174.08	100	1158.32 ± 643.76	6141	6620	863.97 ± 325.59	42873	12732	548.20 ± 181	19352
**COFAR**	1577	413.29 ± 149.78	150	725.25 ± 534.10	129	137	644.16 ± 333.3	455	1271	421.38 ± 207.41	1408

	925708		13008		80961	925708		700019	233158		328476

**Source**: SOLLC: *S. lycopersicum*; SOLPN: *S. pennellii*; SOLHA: *S. habrochaites*; SOLLP: *S. lycopersicum *× *S. pimpinellifolium*; SOLTU: *S. tuberosum*; SOLCH: *S. chacoense*; TOBAC: *N. tabacum*; NICBE: *N. benthamiana*; NICSY: *N. sylvestris*; NICAT: *N. attenuata*; NICLS: *N. langsdorffii *× *N. sanderae*; CAPAN: *C. annuum*; CAPCH: *C. chinense*; PETHY: *Petunia *× *hybrida*; COFCA: *C. canephora*; COFAR: *C. arabica*. **#ESTs**: number of raw ESTs from dbEST; **EST length**: average length and standard deviation; **#mRNA**: number of mRNA from GenBank;**mRNA length**: average length and standard deviation; **#cluster**: number of clusters created by grouping overlapping EST sequences; **#TCs**: number of tentative consensuses which are generated from multiple sequence alignments of ESTs (assembling process); **TC length**: average length and standard deviation; **#ESTs in TCs**: number of ESTs assembled to generate TCs; **#**sESTs:number of singleton ESTs; **sESTs length**: average length and standard deviation;**#Unique transcripts**: number of total transcripts obtained adding the sESTs to the TCs.

### (1) Vector cleaning

RepeatMasker http://repeatmasker.org is used to identify and mask vector sequences by using the NCBI's Vector database (ftp://ftp.ncbi.nih.gov/blast/db/FASTA/vector.gz; update October 2008). The masked regions are removed with an in-house developed trimming tool.

### (2) Repeat masking

EST sequences are masked using the RepeatMasker program with the RepBase.13.06 http://www.girinst.org/ as selected repeat database. Targets for masking include low-complexity regions, simple sequence repeats (SSR, also referred to as microsatellites) and other DNA repeats (e.g. transposable elements).

### (3) Clustering and assembling

For each collection the rate of sequence redundancy was evaluated by first clustering, then assembling EST reads to produce tentative consensus sequences (TCs) and singletons (sESTs; see Table 1). The *wcd *tool [15] was used with its default parameters for the clustering process. The CAP3 assembler [16] with overlap length cutoff (= 60) and an overlap percent identity cutoff (>85) was run to assemble each *wcd *cluster into one or more assembled sets of sequences (i.e. TCs). Indeed, when sequences in a cluster cannot always be all reconciled into a solid and reliable multiple alignment during the assembly process, they are divided into multiple assemblies/TCs. Possible interpretations are: (i) alternative transcription, (ii) paralogy or (iii) protein domain sharing. All the ESTs that did not meet the match criteria to be clustered/assembled with any other EST in the collection were defined as singleton ESTs.

### (4) Annotation

Functional annotation, which is performed both on EST sequences and TCs, is based on the detection of similarities (E-value ≤ 0.001) with proteins by BLAST searches versus the UniProtKB/Swiss-prot (Release 14.3) database. BLAST annotation is detailed including fine-grained gene ontology terms http://www.geneontology.org/ and Enzyme Commission numbers http://www.expasy.ch/enzyme/. A back-end tool to align on-the-fly the unique transcripts against the annotated KEGG-based metabolic pathways http://www.genome.jp/kegg/ was also implemented.

### Database content and web interface

**Figure 1:**
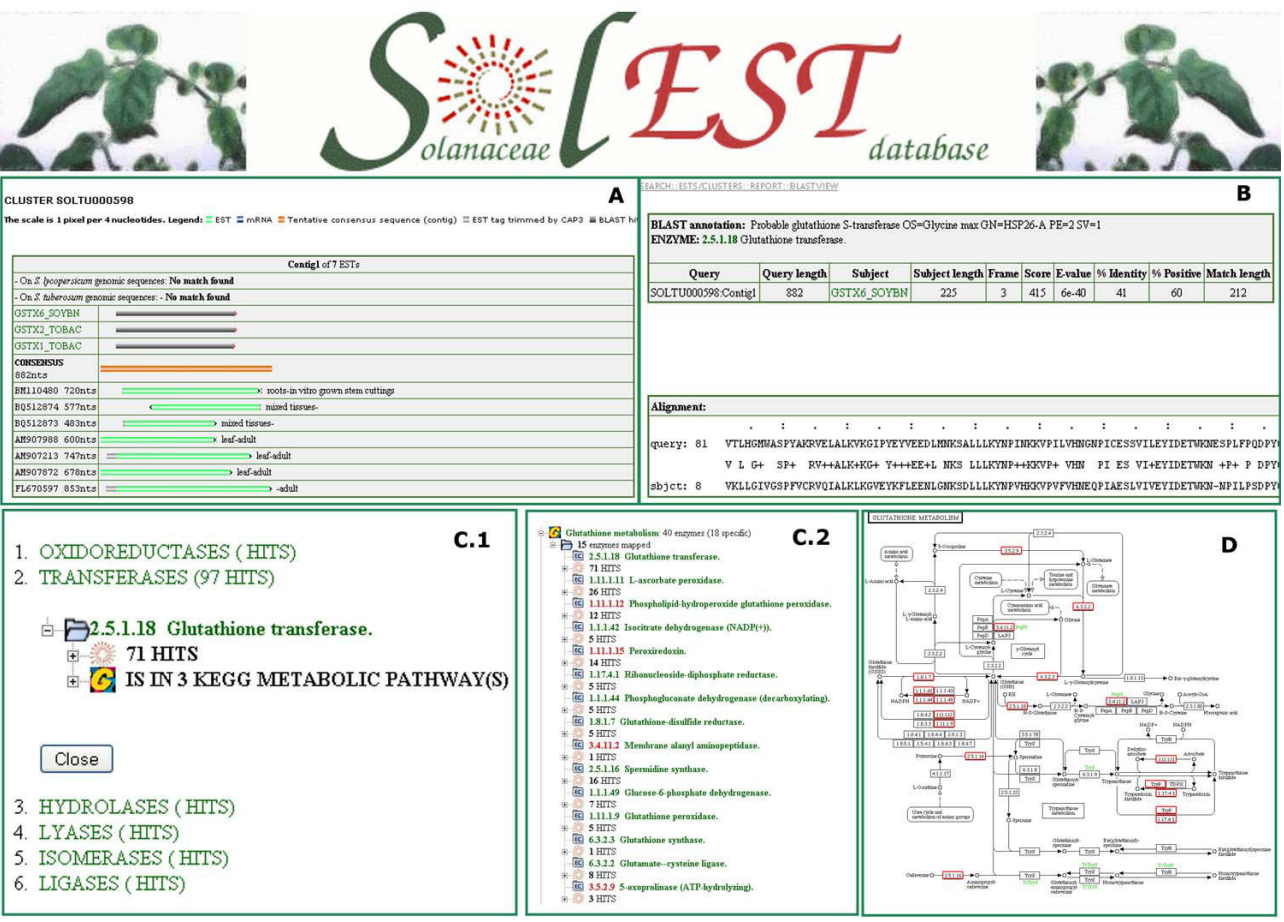
**Snapshots of the SolEST database web interface**. **A**: TC structure and functional annotation. **B**: BLASTx alignment to protein. **C1**: Data classification by ENZYME scheme. **C2**: Data classification by KEGG metabolic pathways. **D**: Transcript association to KEGG metabolic maps.

Raw input ESTs, intermediate data (from the pre-processing analysis) as well as transcript assembly data and annotation information were stored in a MySQL relational database whose structure reproduces the one described in [12]. Web interfaces were implemented in dynamic PHP pages and include Java tree-views for easy object navigation (http://biosrv.cab.unina.it/solestdb/; Figure 1). In addition to well-established access to the EST-based resources via web interfaces, all sequence datasets are available for bulk download in FASTA format in a typical web-based data exchange scenario on the web http://biosrv.cab.unina.it/solestdb/download.php.

## Utility

### Simple sequence repeat (SSR) characterization

**Figure 2:**
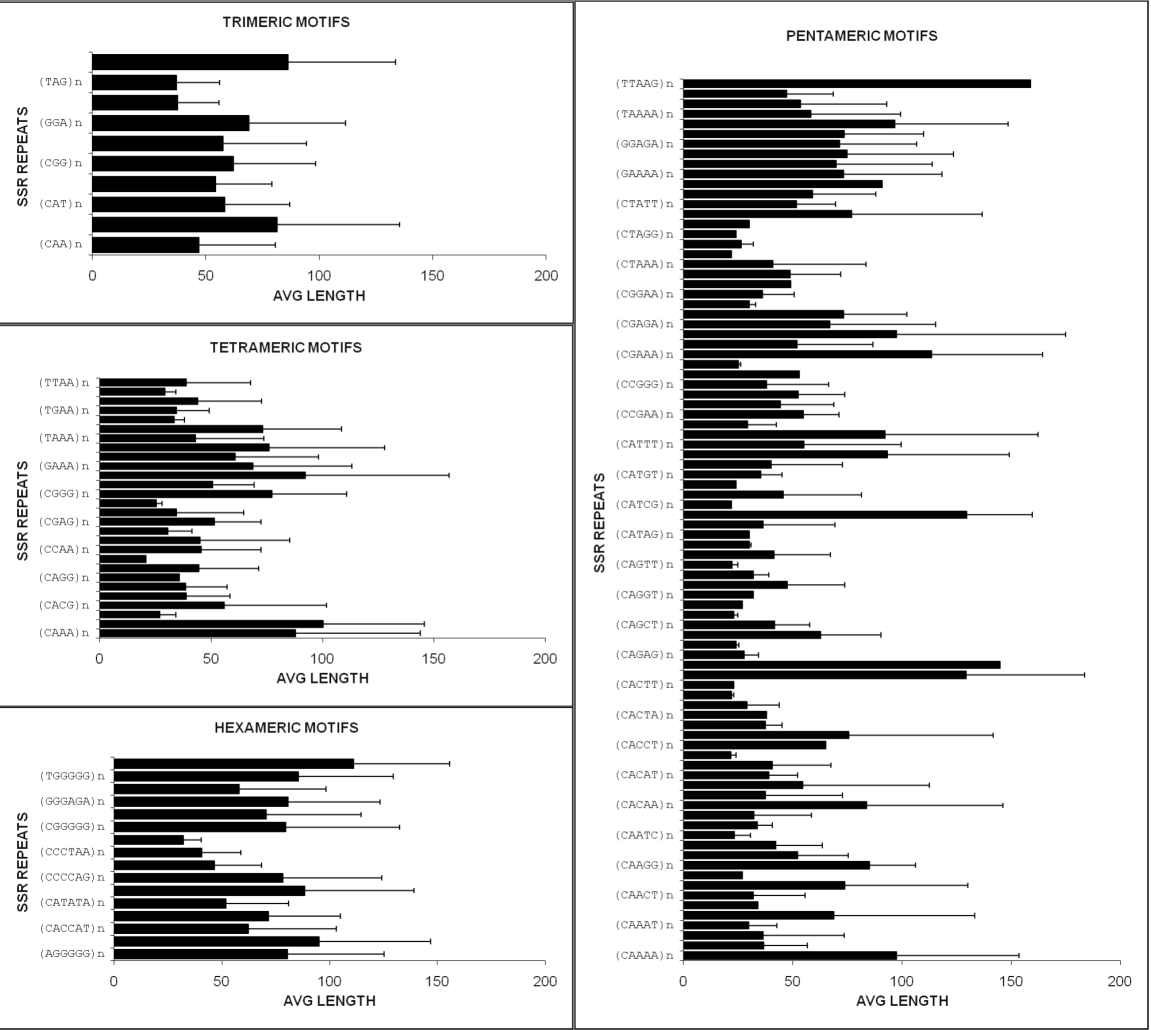
**SSR motif average length**.

EST and mRNA sequences were explored for the existence of microsatellite repeat motifs since they are potential resources for SSR marker discovery [17,18]. Our research focused on trimeric, tetrameric, pentameric and hexameric repeat motifs. In the entire collection we found 10 trimeric, 28 tetrameric, 88 pentameric and 16 hexameric motifs. SSR summary statistics are reported in Table 2, while the frequency of different types of SSR motifs, which were identified species by species, can be found in Additional file 1. In Figure 2, we report the average repeat length and the standard deviation for each SSR motif.

**Table 2:** Simple Sequence Repeats (SSR) summary statistics.

	# sequences analysed	#SSRs identified	# SSR-containing sequences	#sequences containing >1 SSR
**SOLLC**	265760	9636	8758	698
**SOLPN**	8359	360	349	10
**SOLHA**	8030	400	367	27
**SOLLP**	1008	17	15	2
**SOLTU**	232979	12364	10591	1551
**SOLCH**	7812	362	321	30
**TOBAC**	244045	9875	8434	958
**NICBE**	42867	2109	1880	197
**NICSY**	8677	0	0	0
**NICAT**	423	11	11	0
**NICLS**	12543	278	265	10
**CAPAN**	33875	1386	1271	108
**CAPCH**	477	14	13	1
**PETHY**	14340	454	420	27
**COFCA**	55794	3173	2936	200
**COFAR**	1727	142	121	15

**Σ**	938716	40581	35752	3834

**Source**: SOLLC: *S. lycopersicum*; SOLPN: *S. pennellii*; SOLHA: *S. habrochaites*; SOLLP: *S. lycopersicum *× *S. pimpinellifolium*; SOLTU: *S. tuberosum*; SOLCH: *S. chacoense*; TOBAC: *N. tabacum*; NICBE: *N. benthamiana*; NICSY: *N. sylvestris*; NICAT: *N. attenuata*; NICLS: *N. langsdorffii *× *N. sanderae*; CAPAN: *C. annuum*; CAPCH: *C. chinense*; PETHY: *Petunia *× *hybrida*; COFCA: *C. canephora*; COFAR: *C. arabica*.

For each species we show the number of the sequences analysed, the number of the microsatellites identified, the total of the SSR-containing sequences and the amount of sequences containing more than one SSR.

### Comparison of different *Solanaceae *unique transcript sets

We considered the most accessed and referenced *Solanaceae *unigene collections freely available on the web [9-11] in an effort to enable comparisons of different unigene projects for a given species by a comprehensive approach.

Different *Solanaceae *and *Rubiaceae *(genus Coffea) expressed unique transcript sets from the DFCI Gene Index Project (DFCI; http://compbio.dfci.harvard.edu/tgi/plant.html), the plantGDB (PGDB; http://www.plantgdb.org/download/download.php?dir=/Sequence/ESTcontig) and the Solanaceae Genome Network (SGN; ftp://ftp.sgn.cornell.edu/unigene_builds/) were downloaded.

**Figure 3:**
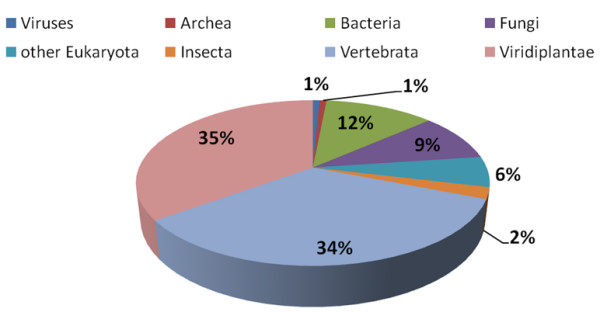
**Pie chart representing protein annotations with respect to the origin of the protein data source**.

In Table 3 the number of collected sequences per species is reported for each of the resources taken into account. Each dataset was compared versus the UniProtKB/Swiss-prot (Release 14.3) database using BLASTX (e-value = 0.001) and the corresponding results are summarized in Table 4. A total of 29,463 distinct proteins were matched corresponding to ~7.35% of the whole protein collection made up of 400,771 sequences. When considering annotations with respect to the origin of the protein data source, the bulk of the identifications concerned proteins of plant and vertebrata origin (35% and 34%, respectively), while protein from bacteria and fungi represent 12% and 9% as reported in figure 3. We built a web tool dedicated to enable the association of different unigene collections for a given *Solanaceae *species based on the UniProt Knowledgebase annotations. Data can be accessed by specifying the UniProt accession number, the UniProt entry name or keywords; the latter may be searched in the protein description lines http://biosrv.cab.unina.it/solestdb/solcomp.php.

**Table 3:** Number of sequences per species collected from different web sources.

	TOTAL UNIQUE SEQUENCES		
**SOURCE**	**DFCI**	**PlantGDB**	**SGN**

**SOLLC**	46849	48945	34829
**SOLPN**		3718	
**SOLHA**		4024	
**SOLTU**	?	70344	31072
**SOLCH**		7110	
**TOBAC**	83083	114188	84602
**NICBE**	16127	18037	16024
**NICSY**		7612	6300
**NICLS**		6791	
**CAPAN**	14249	15278	9554
**PETHY**	8729	9884	5135
**COFCA**	17632	20168	15721
**COFAR**		1093	

**Source**: SOLLC: *S. lycopersicum*; SOLPN: *S. pennellii*; SOLHA: *S. habrochaites*; SOLLP: *S. lycopersicum *× *S. pimpinellifolium*; SOLTU: *S. tuberosum*; SOLCH: *S. chacoense*; TOBAC: *N. tabacum*; NICBE: *N. benthamiana*; NICSY: *N. sylvestris*; NICAT: *N. attenuata*; NICLS: *N. langsdorffii *× *N. sanderae*; CAPAN: *C. annuum*; CAPCH: *C. chinense*; PETHY: *Petunia *× *hybrida*; COFCA: *C. canephora*; COFAR: *C. arabica*.**CAB**: Computer Aided Bioscience group http://cab.unina.it collection; **DFCI**: The DFCI Gene Index Project http://compbio.dfci.harvard.edu/tgi/; **PGDB**: PlantGDB http://www.plantgdb.org/; **SGN**: The unigene collection at Solanaceae Genomics Network http://www.sgn.cornell.edu/. '**?**' indicates that the corresponding sequence file was corrupted at the time of the analysis.

**Table 4:** Statistics on UniProtKB-based annotations.

Unique transcripts with matches in UniProt				
**SOURCE**	**CAB**	**DFCI**	**PGDB**	**SGN**

**SOLLC**	28737 (55.82%)	27240 (58.1%)	27763 (56.7%)	20720 (59.4%)
**SOLPN**	2319 (58.65%)	-	2169 (58.3%)	-
**SOLHA**	2652 (67.14%)	-	2690 (66.8%)	-
**SOLLP**	483 (68.71%)	-	-	-
**SOLTU**	39201 (54.38%)	-	37920 (53.9%)	17122 (55.1%)
**SOLCH**	4163 (60.19%)	-	4062 (57.1%)	
**TOBAC**	45647 (41.5%)	30958 (37.26%)	46618 (40.83%)	33415 (39.5%)
**NICBE**	8108 (43.15%)	7631 (47.32%)	8564 (47.48%)	7239 (45.18%)
**NICSY**	3930 (52.3%)	-	4030 (52.9%)	3512 (55.7%)
**NICAT**	177 (46.09%)	-	-	-
**NICLS**	3116 (49.09%)		3391 (49.9%)	-
**CAPAN**	8457 (52.83%)	7947 (55.7%)	8454 (55.3)%	5723 (59.90%)
**CAPCH**	311 (73.52%)	-	-	-
**PETHY**	5041 (53.9%)	4999 (57.27%)	5474 (55.3%)	2967 (57.7%)
**COFCA**	9896 (51.14%)	9464 (53.6%)	10697 (53.0%)	8316 (52.9%)
**COFAR**	701 (49.79%)	-	530 (48.49%)	-

The table shows the number of sequences with significant matches to the UniProtKB/Swiss-prot database and, in brackets, the corresponding percentage on the total.

**Source**: SOLLC: *S. lycopersicum*; SOLPN: *S. pennellii*; SOLHA: *S. habrochaites*; SOLLP: *S. lycopersicum *× *S. pimpinellifolium*; SOLTU: *S. tuberosum*; SOLCH: *S. chacoense*; TOBAC: *N. tabacum*; NICBE: *N. benthamiana*; NICSY: *N. sylvestris*; NICAT: *N. attenuata*; NICLS: *N. langsdorffii *× *N. sanderae*; CAPAN: *C. annuum*; CAPCH: *C. chinense*; PETHY: *Petunia *× *hybrida*; COFCA: *C. canephora*; COFAR: *C. arabica*.**CAB**: Computer Aided Bioscience group http://cab.unina.it collection; **DFCI**: The DFGI Gene Index Project http://compbio.dfci.harvard.edu/tgi/; **PGDB**: Plant Genome Database http://www.plantgdb.org/; **SGN**: The unigene collection at Solanaceae Genomics Network http://www.sgn.cornell.edu/).

**Figure 4:**
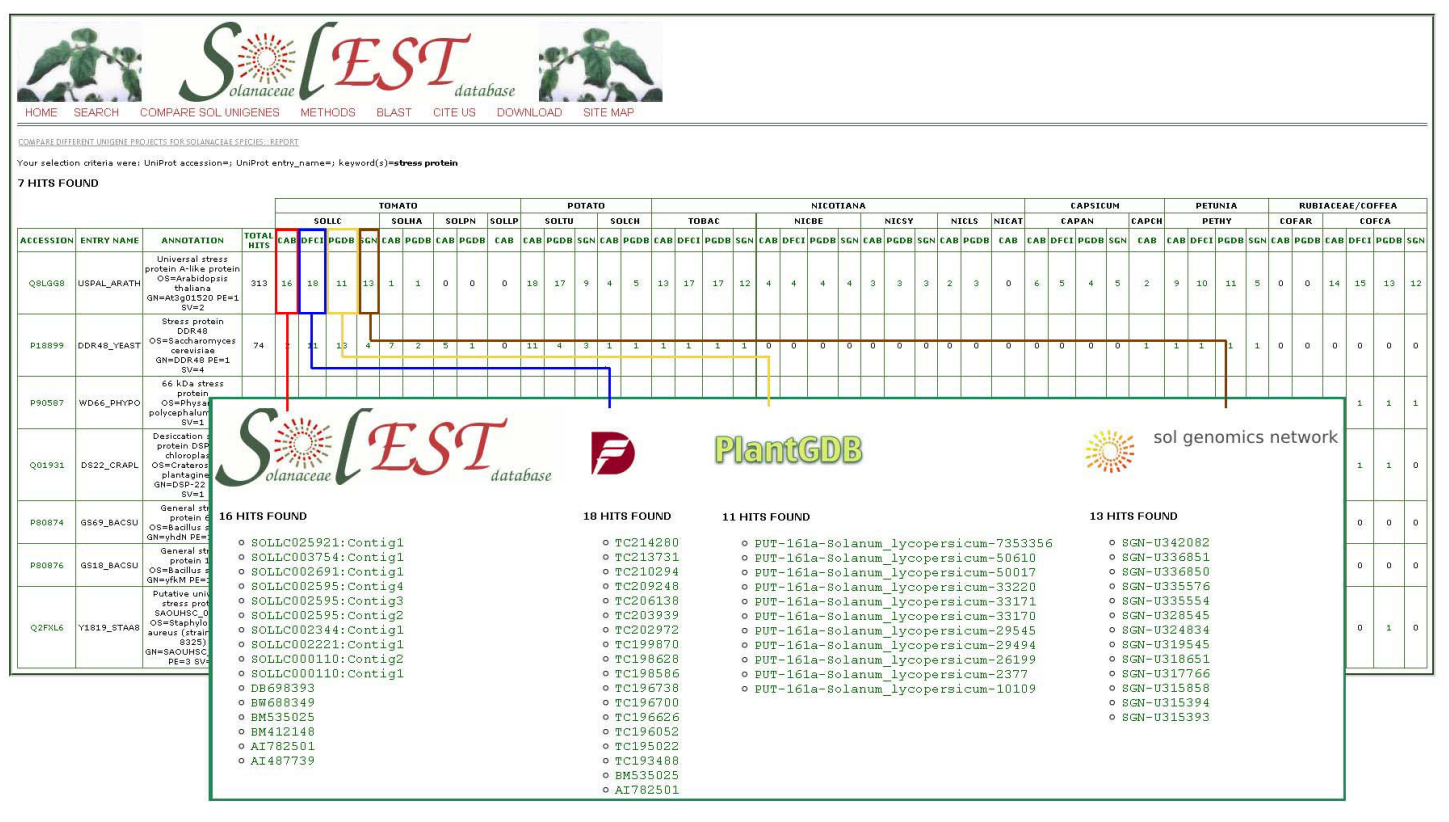
**Screenshot of the web tool for comparing different unigene collections for a given Solanaceae species**. Panel A shows results from a query in matrix format where each row represents a protein from the UniProt Knowledgebase database and each column refers to a single *Solanaceae *species and unigene collection. Each matrix cell defines the number of unique transcripts matching a protein sequence. By clicking on a single matrix cell the user can access the list of source-specific sequence identifiers (Panel B).

The results of a query are displayed in *matrix *format where each row represents a protein and each column refers to a single species for each web resource. The (i, j)th entry of the matrix identifies the number of unique transcripts matching a protein sequence (Figure 4A). By clicking on a single matrix cell the user can access the list of source-specific sequence identifiers (Figure 4B), each of which is, in turn, used to generate a cross-reference to the SolEST database itself as well as to the corresponding external database.

### Exploiting SolEST for *Solanaceae *genome sequencing

EST-based collections represent a much-needed reference for the structural annotation of the emerging *Solanaceae *genome sequences and for addressing sequence-based synteny studies. In addition, they can support technical issues arising while sequencing efforts are ongoing.

1,215 BAC sequences from *S. lycopersicum *and 708 from *S. tuberosum *were retrieved from GenBank on July 2009. ESTs and TC sequences from tomato and potato were spliced-aligned along BAC sequences using GenomeThreader [19]. Alignments with a minimum score identity of 90% and a minimum sequence coverage of 80% were filtered out.

Table 5 shows the number of ESTs and TCs per species successfully mapped along the available BAC sequences from tomato and potato (see Methods).

**Table 5:** Counting of ESTs/TCs mapped along tomato and potato genomic sequences.

		mapped on TOMATO			mapped on POTATO			mapped on TOMATO and/or POTATO		
**SOURCE**	**TOTAL (ESTs/TCs)**	**# total (ESTs/TCs)**	**# multiple matches (ESTs/TCs)**	**# single matches (ESTs/TCs)**	**# total (ESTs/TCs)**	**# multiple matches (ESTs/TCs)**	**# single matches (ESTs/TCs)**	**only TOMATO (ESTs/TCs)**	**only POTATO (ESTs/TCs)**	**TOMATO & POTATO (ESTs/TCs)**

**CAPAN**	33311/4293	3015/365	805/89	2210/276	1051/117	258/24	793/93	2585/307	621/59	430/58

**CAPCH**	372/34	41/3	20/3	21/0	23/2	3/1	20/1	35/3	17/2	6/

**COFAR**	1577/137	30/3	10/1	20/2	30/3	8/0	22/3	1/3	1/	29/3

**COFCA**	55694/6620	73/7	51/6	22/1	58/7	42/5	16/2	22/1	7/1	51/6

**NICAT**	329/32	16/0	8/0	8/0	14/0	4/0	10/0	7/0	5/0	9/0

**NICBE**	42566/5006	1016/80	363/20	653/60	499/37	102/11	397/26	764/60	247/17	252/20

**NICLS**	12448/1379	207/27	64/7	143/20	106/10	37/1	69/9	163/23	62/6	44/4

**NICSY**	8583/674	546/52	140/17	406/35	215/19	49/5	166/14	464/46	133/13	82/6

**PETHY**	14017/1738	37/278	12/64	25/214	12/119	1/22	11/97	33/227	8/68	4/51

**SOLCH**	7752/637	1068/117	262/29	806/88	469/58	107/10	362/48	925/103	326/44	143/14

**SOLHA**	8000/1243	1996/346	658/99	1338/247	600/99	194/15	406/84	1786/306	390/59	210/40

**SOLLC**	259990/20548	86547/6184	24129/1576	62418/4608	22126/1409	4985/299	17141/1110	76161/5485	11740/710	10386/699

**SOLLP**	1008/109	42/0	13/0	29/0	9/0	2/0	7/0	39/0	6/0	3/0

**SOLPN**	8346/844	2731/269	766/77	1965/192	772/66	100/14	672/52	2425/240	466/37	306/29

**SOLTU**	231275/23453	48264/4314	12936/1096	35328/3218	22189/1974	5454/458	16735/1516	41371/3693	15296/1353	6893/621

**TOBAC**	240440/28571	8171/499	2310/123	5861/376	3686/247	977/58	2709/189	6516/392	2031/140	1655/107

The total number of ESTs/TCs for each *Solanaceae *species collected in the SolEST database is shown in the first two columns. The number of ESTs/TCs splice-aligned along BACs, the number of ESTs/TCs mapped more than once and the number of EST/TC single matches is reported for the tomato and potato genomes, respectively. In addition, the table lists the number of ESTs/TCs exclusively mapped onto the tomato or potato genome and, finally, the number of ESTs/TCs splice-aligned along both the genomes.

We estimated the level of coverage of the *Solanaceae *transcriptome by counting the number of ESTs/TCs mapped with respect to the total number of the sequences collected in SolEST. The different transcriptome coverage per species is informative *per se *of the similarity level of the *Solanaceae *transcriptomes. For example, the EST/TC dataset from tobacco (*Nicotiana tabacum*), even if it is solid in number, proved poorly mapped on both tomato and potato BACs, showing a transcriptome distance with respect to *S. lycopersicum*, *S. tuberosum *or *C. annuum*.

Columns 4 and 7 in Table 5 report the number of ESTs/TCs with multiple matches along tomato as well as potato BACs. This is expected since sequencing proceeds on a BAC-by-BAC basis, aiming at a minimal tiling path of BACs. In other words, it is evident that several transcripts are aligned along different BACs of the same chromosome because of BAC overlaps. As an alternative, transcripts with multiple matches can be identified with repetitive sequences in the genomes.

Table 6 shows that concurrent mapping of *Solanaceae *ESTs/TCs along the tomato and potato BAC sequences is informative not only for investigating genome co-linearity between the two species but also for supporting genome sequencing and assignment of BACs to the corresponding chromosomes.

**Table 6:** Examples of *S. lycopersicum *TCs that are independently splice-aligned along tomato and potato BACs.

			S.lycopersicum BACs				S.tuberosum BACs			
			
	TC ID	UniProtKB Annotation	# chr	BAC ID	start	stop	# chr	BAC ID	start	stop
**A**	SOLLC004853:Contig2		1	AC171727.1	13505	14336	-	-	-	-
	
	SOLLC004853:Contig1		1	AC171727.1	13540	14538	-	-	-	-
	
	SOLLC005165:Contig75	Q8L9T5 | ATL3F_ARATH | RING-H2 finger protein ATL3F OS = *Arabidopsis thaliana*	1	AC171727.1	36486	37772	-	-	-	-
	
	SOLLC007669:Contig1	Q43043 | PME_PETIN | Pectinesterase OS = *Petunia integrifolia*	1	AC171727.1						
	
	SOLLC021190:Contig1	Q43043 | PME_PETIN | Pectinesterase OS = *Petunia integrifolia*	1	AC171727.1	109415	109926	-	-	-	-
	
	SOLLC020772:Contig1	Q43043 | PME_PETIN | Pectinesterase OS = *Petunia integrifolia*	1	AC171727.1	121192	121569	-	-	-	-
	
	SOLLC015580:Contig1	Q43043 | PME_PETIN | Pectinesterase OS = *Petunia integrifolia*	1	AC171727.1	121518	123321	-	-	-	-

**B**	SOLLC004826:Contig1	Q766C2 | NEP2_NEPGR | Aspartic proteinase nepenthesin-2 OS = *Nepenthes gracilis*	-	-	-	-	5	AC233494.1	5393	6182
	
	SOLLC021577:Contig1	Q766C3 | NEP1_NEPGR | Aspartic proteinase nepenthesin-1 OS = *Nepenthes gracilis*	-	-	-	-	5	AC233494.1	6647	7214
	
	SOLLC005426:Contig1		-	-	-	-	5	AC233494.1	10450	13585
	
	SOLLC007282:Contig1	Q9FT81 | TT8_ARATH | Protein TRANSPARENT TESTA 8 OS = *Arabidopsis thaliana*	-	-	-	-	5	AC233494.1	34176	35673
	
	SOLLC012069:Contig1	Q94HW3 | DRL11_ARATH | Probable disease resistance protein RDL6/RF9 OS = *Arabidopsis thaliana*	-	-	-	-	5	AC233494.1	48246	53485
	
	SOLLC024651:Contig1	Q6L400 | R1B16_SOLDE | Putative late blight resistance protein homolog R1B-16 OS = *Solanum demissum*	-	-	-	-	5	AC233494.1	51418	52370
	
	SOLLC010379:Contig1	Q38950 | 2AAB_ARATH | Serine/threonine-protein phosphatase 2A 65 kDa regulatory subunit A beta isoform OS = *Arabidopsis thaliana*	-	-	-	-	5	AC233494.1	71687	79002
	
	SOLLC024982:Contig1		-	-	-	-	5	AC233494.1	84414	87845

**C**	SOLLC010896:Contig1		11	AC212431.2	128060	132631	0	AC232103.1	40703	46333
	
	SOLLC005049:Contig1	Q3ZAF9 | KGUA_DEHE1 | Guanylate kinase OS = *Dehalococcoides ethenogenes*	11	AC212431.2	137482	138460	0	AC232103.1	33385	34367
	
	SOLLC032933:Contig1	P54677 | PI4K_DICDI | Phosphatidylinositol 4-kinase OS = *Dictyostelium discoideum*	11	AC212431.2	159306	163292	0	AC232103.1	13520	17677

**D**	SOLLC001795:Contig1	acc = P43394 entry_name = K502_ACTDE desc = Fruit protein pKIWI502 OS = *Actinidia deliciosa*	0	CU914756.3	126304	131068	5	AC233527.1	81221	85811
	
	SOLLC001995:Contig1	acc = P17614 entry_name = ATPBM_NICPL desc = ATP synthase subunit beta, mitochondrial OS = *Nicotiana plumbaginifolia*	0	CU914756.3	121815	125827	5	AC233527.1	75747	79701
	
	SOLLC002780:Contig1		0	CU914756.3	132958	137854	5	AC233527.1	87980	93448
	
	SOLLC002780:Contig2		0	CU914756.3	132996	137857	5	AC233527.1	88066	93451
	
	SOLLC011112:Contig1	acc = P54086 entry_name = Y194_SYNY3 desc = Uncharacterized protein sll0194 OS = *Synechocystis*	0	CU914756.3	68182	73146	5	AC233527.1	56111	61611
	
	SOLLC014928:Contig1	acc = P34552 entry_name = ALX1_CAEEL desc = Apoptosis-linked gene 2-interacting protein X 1 OS = *Caenorhabditis elegans*	0	CU914756.3	141248	142048	5	AC233527.1	96451	97251

**E**	SOLLC013836:Contig1		11	AC171736.1	46790	49549	11	AC231674.1	1	2996
	
	SOLLC033755:Contig1	Q5T9S5 | CCD18_HUMAN | Coiled-coil domain-containing protein 18 OS = *Homo sapiens*	11	AC171736.1	61542	62195	11	AC231674.1	19771	20423
	
	SOLLC011109:Contig2	Q9FIH9 | CML37_ARATH | Calcium-binding protein CML37 OS = *Arabidopsis thaliana*	11	AC171736.1	67251	68067	11	AC231674.1	29350	30116
	
	SOLLC027329:Contig1	Q7Z2Z2 | ETUD1_HUMAN | Elongation factor Tu GTP-binding domain-containing protein 1 OS = *Homo sapiens*	11	AC171736.1	71740	72558	11	AC231674.1	34634	35449
	
	SOLLC011285:Contig1		11	AC171736.1	78845	82124	11	AC231674.1	46882	50643
	
	SOLLC000854:Contig1	Q9D7N9 | APMAP_MOUSE | Adipocyte plasma membrane-associated protein OS = *Mus musculus*	11	AC171736.1	82643	87091	11	AC231674.1	50695	55627
	
	SOLLC016421:Contig1	Q6R2K2 | SRF4_ARATH | Protein STRUBBELIG-RECEPTOR FAMILY 4 OS = *Arabidopsis thaliana*	11	AC171736.1	101708	106816	11	AC231674.1	72838	78011
	
	SOLLC004514:Contig1	P42824 | DNJH2_ALLPO | DnaJ protein homolog 2 OS = *Allium porrum*	11	AC171736.1	107145	110458	11	AC231674.1	78559	81871
	
	SOLLC014445:Contig1	Q9SF32 | IQD1_ARATH | Protein IQ-DOMAIN 1 OS = *Arabidopsis thaliana*	11	AC171736.1	119739	122235	11	AC231674.1	97126	101323
	
	SOLLC026		11	AC17	12536	12605	11	AC23	10606	10674
	
	122:Contig1			1736.1	1	5		1674.1	6	4
	
	SOLLC013	P16577 | UBC4_WHEAT |	11	AC17	13590	13989	11	AC23	11693	12091
	
	099:Contig4	Ubiquitin-conjugating enzyme E2-23 kDa OS = *Triticum aestivum*		1736.1	0	2		1674.1	4	9

**F**	SOLLC014596:Contig1		4	CU914524.3	11054	11642	1	AC233501.1	124622	125217
	
	SOLLC004364:Contig1	Q9SZA7 | DRL29_ARATH | Probable disease resistance protein At4g33300 OS = *Arabidopsis thaliana*	4	CU914524.3	19738	21366	1	AC233501.1	115958	117638
	
	SOLLC003401:Contig2	Q9SZA7 | DRL29_ARATH | Probable disease resistance protein At4g33300 OS = *Arabidopsis thaliana*	4	CU914524.3	21414	22905	1	AC233501.1	114425	115910
	
	SOLLC003654:Contig1	Q8GZD4 | NAT3_ARATH | Nucleobase-ascorbate transporter 3 OS = *Arabidopsis thaliana*	4	CU914524.3	24161	31350	1	AC233501.1	105840	113085
	
	SOLLC009218:Contig1	Q9S7T8 | SPZX_ARATH | Serpin-ZX OS = *Arabidopsis thaliana*	4	CU914524.3	34754	37499	1	AC233501.1	100513	103214
	
	SOLLC010320:Contig1	Q9S7T8 | SPZX_ARATH | Serpin-ZX OS = *Arabidopsis thaliana*	4	CU914524.3	39986	41937	1	AC233501.1	79650	81701
	
	SOLLC005339:Contig2	Q9S7T8 | SPZX_ARATH | Serpin-ZX OS = *Arabidopsis thaliana*	4	CU914524.3	43123	43880	1	AC233501.1	77665	78422
	
	SOLLC007010:Contig1	Q05085 | CHL1_ARATH | Nitrate/chlorate transporter OS = *Arabidopsis thaliana*	4	CU914524.3	52979	54985	1	AC233501.1	40918	42609
	
	SOLLC005747:Contig1	Q05085 | CHL1_ARATH | Nitrate/chlorate transporter OS = *Arabidopsis thaliana*	4	CU914524.3	56811	57920	1	AC233501.1	41189	42544
	
	SOLLC008759:Contig1		4	CU914524.3	60617	63084	1	AC233501.1	30324	32692
	
	SOLLC002208:Contig1	O04348 | TPP1_ARATH | Thylakoidal processing peptidase 1, chloroplastic OS = *Arabidopsis thaliana*	4	CU914524.3	63803	67165	1	AC233501.1	25016	29581
	
	SOLLC004022:Contig1		4	CU914524.3	74867	75517	1	AC233501.1	12497	13142

Each selected TC is identified by its ID (TC ID), and a putative functional annotation is associated to it (UniProtKB Annotation). In case of TCs aligned along *S. lycopersicum *and/or *S. tuberosum *BACs, the chromosome number (# chr), the BAC accession number from GenBank (BAC ID), and the BAC start and stop coordinates are reported.

First of all, panels A and B in table 6 report instances of *S. lycopersicum *TCs solely mapped on BACs from a unique species. In particular, 5,904 *S. lycopersicum *TCs mapped exclusively on tomato genome sequences, while 488 were successfully aligned only along potato BACs, suggesting that the potato sequencing project, although started later, is providing a complementary contribution to that of tomato.

Tomato as well as potato BACs with ambiguous positioning on chromosomes, which have been assigned to the arbitrary-defined chromosome 0, can be correctly associated to the corresponding chromosomes by exploiting (Table 6, panels C and D) evidence from the potato or tomato counterpart, respectively.

In most cases, it is useful to refer to a comparison of BAC sequences, while they are released, in an attempt to find clear genome co-linearity with tomato/potato (Table 6 panels E) or to highlight neighboring genetic *loci *which retain their relative positions and orders on different chromosomes of the two species (Table 6 panel F).

**Figure 5:**
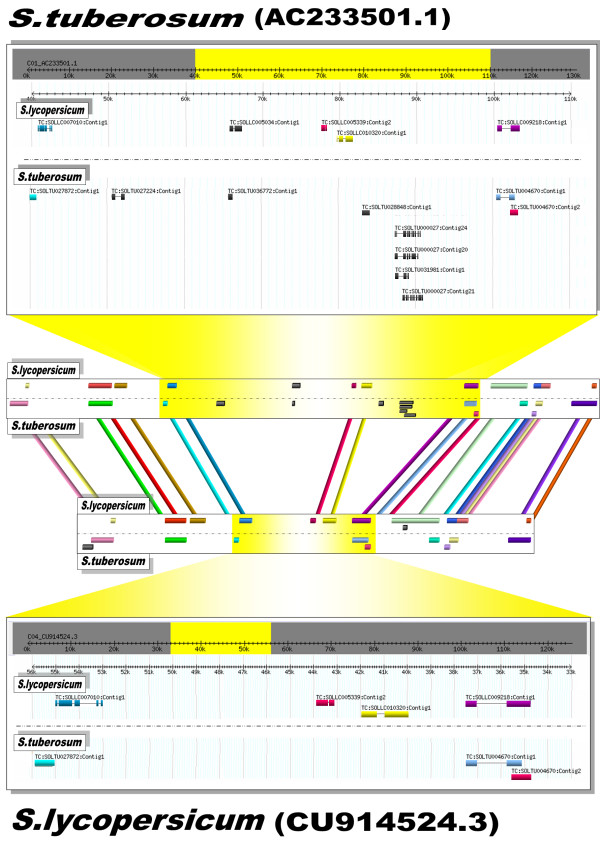
**Representation of the *co-linearity *between the tomato BAC CU914524.3 and the potato BAC AC233501.1**. The BAC CU914524.3 from tomato and the BAC AC233501.1 from potato are present schematically at the center of the figure. Transcripts from *S. lycopersicum *and *S. tuberosum *mapped onto them are represented as colored bars (same colors identified same TCs). A particular genomic region is emphasized in yellow and is "zoomed-in" in order to display details on the TC splice-alignments.

Figure 5 shows an example that points to the power of a comparative approach based on different transcriptome and genome collections integrated in a single platform. Transcripts from *S. lycopersicum *and *S. tuberosum *were mapped onto the BAC CU914524.3 from tomato and the BAC AC233501.1 from potato. The two BACs are present schematically at the center of the figure and were selected because they share a remarkable number of TCs (20 TCs) which are represented as colored bars (the same colors identified the same TCs). Clearly, all the TCs successfully aligned along the BAC CU914524.3 are mapped onto the potato BAC AC233501.1 maintaining their relative positions and orders. It can be easily assumed that the two genomic regions taken into account are co-linear. However, they differ in size, the potato genomic region being 120 kb and that of tomato 70 kb. This is due to insertions in the potato BAC. In these inserted regions TCs from both the species are present (black bars). The region which we are describing is highlighted in yellow and is "zoomed-in" in order to display details on the TC splice-alignments.

SolEST currently provides information on the mapping of both EST and TC datasets on the draft sequences of the tomato and potato genome in the framework of platform ISOL@ [13].

## Discussion

Different databases worldwide are related in concept, content and utility to the one presented herein. All of them aim to partition EST sequences into a non-redundant set of gene-oriented clusters and to provide sequences with related information such as biological function and the tissue types in which the gene is expressed. Of course, they differ in their database update policy, in data quality standards and finally in the level of detail with which the database is endowed, which supports investigations on structural and functional information and on expression patterns to different extents.

The SolEST database presents several features making it appealing for the SOL research community and for those interested in EST data management. The *Solanaceae *EST collection is endowed with both immediate graphical interfaces and details on the organization of multiple alignments and consensus sequence structure to permit a user friendly interpretation of the results as well as easy access to accessory information. SolEST can be accessed through different access points which are briefly summarized to describe the main features of the database that were, however, inherited by TomatEST [12]. The 'Unique Transcript' access point allows the list of singletons and tentative consensus sequences to be associated to the enzymes they encode and, as a consequence, to be mapped 'on the fly' on the KEGG-based metabolic pathways. Singleton ESTs as well as ESTs which were assembled generating the corresponding TC can be independently browsed through the '*ESTs*' access point. The maintenance of the single ESTs as well as of the background information related to each of them (presence of contamination and of repeat subsequences, functional annotation), makes SolEST suitable for accessing raw data also in the event of updating the database. This represents an attractive feature of TomatEST [12] which was saved in SolEST. Finally, the '*cluster*' access point allows those clusters which have been split into multiple assemblies to be browsed. It can be exploited for *a priori *identification of putative alternative transcripts or allele-specific transcript isoforms and for investigation of heterozygosity and on the level of ploidy of many of the included species

Being in possession of the entire publicly available EST collection for *Solanaceae *permitted identification of SSR and the building of a comprehensive EST-derived SSR catalogue for *Solanaceae *which is accessible to users. This catalogue can be used to develop genetic markers, opening up additional paths into *Solanaceae *phylogenetic and evolutionary analysis and genetic mapping.

Another novel feature of the SolEST database is aimed at resolving a common complaint in the SOL community as to whether different *Solanaceae *assemblies generated by diverse research groups should be compared. Various clustering and assembly programs or parameters result in differences among the unique transcript sets provided by different reference databases. In addition, given that each set can be built starting from non-homogeneous primary data sources (e.g. dbEST, RefSeq, genomic or unfinished high-throughput cDNA sequencing (HTC) entries), differences can further become larger. The different major collections now available for the *Solanaceae *transcriptomes are however equivalently used by the entire community, as they represent the basic collection for specific expression arrays (e.g. a list of microarray resources for tomato is available in [20] Table 2), for COS marker definition [11], for genome annotation [12]. In order to overcome such differences, we decided to compare each unigene collection with the UniProt Knowledgebase. The use of a protein reference database may represent a useful tool to cross-link the different collections through a specific service and, more interestingly, it is an immediate approach to compare the different EST-based available resources.

One of the most novel features in SolEST, when compared to other resources, is the option of accessing and then visualizing *Solanaceae *EST/TC alignments along the tomato and the potato genomes. The mapping of *Solanaceae *ESTs certainly provides insights into the location of potential candidate genes and facilitates EST-driven gene annotation. This represents the first attempt to provide a unique view of the data from both the sequencing efforts, which we believe will be appreciated by the SOL community. In addition, having ESTs from *Solanaceae *and *Rubiaceae *mapped along the genomes of two of the major representatives of the family will support comparative genomics approaches aimed at addressing the most fundamental issues such as diversity and adaptation within the *Solanaceae *family and heterogeneity in gene expression patterns. Finally, the TCs we defined will provide support for solving technical issues arising from BAC-by-BAC genome sequencing and will undoubtedly provide a reference for forthcoming WGS (whole genome shotgun) efforts in both tomato and potato.

## Conclusion

To our knowledge, no similar work has yet been carried out on the construction of an EST database offering a broad overview of *Solanaceae *as well as *Coffea *transcriptomes. Multiple sequence analysis results from the database (e.g. developing a unigene set, annotation with putative function and identification of SSRs) extensively linked to external related resources, represent a major source of information for these plant families, opening up novel vistas conducive to comparative evolutionary studies. We think the SolEST database will represent an invaluable resource for supporting the structural annotation of the emerging *Solanaceae *genome sequences and addressing technical issues arising while sequencing efforts are being made. Finally, the SolEST database meets the challenge of connecting the different EST-centered collections worldwide generated by applying various methods and starting from disparate primary data sources.

## Availability and requirements

The SolEST database is available with no restrictions at the following URL: http://biosrv.cab.unina.it/solestdb/.

The SolEST update is scheduled at the end of each year and comprises the retrieval of primary data sources (i.e. EST/mRNA sequences) and the generation of novel unigenes/TCs as well as their annotation. Therefore, at each release the update of the satellite databases (i.e. UniVec, RepBase, UniProtKB/Swiss-prot, Gene Ontology, Enzyme, KEGG) used in the cleaning, repeat masking and annotation phases is also performed.

The retrieval of new *S. lycopersicum *and *S. tuberosum *BAC sequences from the GenBank repository is ensured daily by an automated pipeline [13]. The switch to genome contigs will beensured as the sequencing status will evolve.

## Authors' contributions

NDA was mainly involved in the development, organization and maintenance of the SolEST database and wrote the manuscript; AT was involved in setting up the comparative analysis of the genome sequences from tomato and potato; LF contributed to carrying out the project; MLC conceived the project, directed its design and implementation, coordinated the different efforts and wrote the manuscript. All authors read and approved the final manuscript.

## Supplementary Material

Additional file 1 **Frequency of SSR in each species-specific EST collection**. The 142 SSR motifs we identified are listed and grouped according to their unit size. For each motif the observed frequency in each species-specific collection is reported. SOLLC: *S. lycopersicum*; SOLPN: *S. pennellii*; SOLHA: *S. habrochaites*; SOLLP: *S. lycopersicum *× *S. pimpinellifolium*; SOLTU: *S. tuberosum*; SOLCH: *S. chacoense*; TOBAC: *N. tabacum*; NICBE: *N. benthamiana*; NICSY: *N. sylvestris*; NICAT: *N. attenuata*; NICLS: *N. langsdorffii *× *N. sanderae*; CAPAN: *C. annuum*; CAPCH: *C. chinense*; PETHY: *Petunia *× *hybrida*; COFCA: *C. canephora*; COFAR: *C. arabica*. Click here for file
